# MMP-9 as a Candidate Marker of Response to BRAF Inhibitors in Melanoma Patients With *BRAF*^V600E^ Mutation Detected in Circulating-Free DNA

**DOI:** 10.3389/fphar.2018.00856

**Published:** 2018-08-14

**Authors:** Rossella Salemi, Luca Falzone, Gabriele Madonna, Jerry Polesel, Diana Cinà, Domenico Mallardo, Paolo A. Ascierto, Massimo Libra, Saverio Candido

**Affiliations:** ^1^Department of Biomedical and Biotechnological Sciences, University of Catania, Catania, Italy; ^2^Unit of Melanoma, Cancer Immunotherapy and Innovative Therapy, Istituto Nazionale Tumori “Fondazione G. Pascale”, Naples, Italy; ^3^Unit of Cancer Epidemiology, Aviano National Cancer Institute, IRCCS, Aviano, Italy; ^4^Clinical Pathology Unit, Garibaldi Hospital, Catania, Italy; ^5^Research Center for Prevention, Diagnosis and Treatment of Cancer, University of Catania, Catania, Italy

**Keywords:** *BRAF^V600E^*, MMP-9, circulating-free DNA, biomarker, droplet digital PCR, liquid biopsy

## Abstract

The *BRAF^V600E^* mutation is associated with melanoma development and its detection in circulating-free DNA cannot be observed in all melanoma patients harboring this mutation in tumor specimens. Beside the circulating-free DNA *BRAF^V600E^* mutation, other markers of therapeutic response should be identified. Matrix metalloproteinase-9 (MMP-9) could be one of them as its role as indicator of invasiveness in melanoma have been explored. In this study, MMP-9 was evaluated in melanoma cells after treatment with dabrafenib. *In vitro* data were validated in 26 melanoma patients, of which 14 treated with BRAF inhibitor alone and 12 treated with both BRAF and MEK inhibitors, by ELISA assay and droplet digital PCR for measuring MMP-9 serum levels and circulating-free DNA *BRAF^V600E^* mutation, respectively. Statistical analyses were performed to evaluate the prognostic significance of MMP-9, progression-free survival (PFS) and overall survival (OS) according to the *BRAF^V600E^* mutation and MMP-9 levels. The performed analyses showed that MMP-9 and pEKR1-2 were statistically down-regulated in melanoma cells after treatment with dabrafenib. Circulating-free DNA *BRAF^V600E^* mutation was detected in 11 out of 26 melanoma patients showing higher levels of MMP-9 compared to those with undetectable *BRAF^V600E^* mutation. Furthermore, higher levels of MMP-9 and circulating-free DNA *BRAF^V600E^* mutation were associated with lower PFS and OS. Finally, the monitoring of therapy showed that MMP-9 significantly decreased at T1 and T2, but not at T-last, for the patients with detectable circulating-free DNA *BRAF^V600E^* mutation. In conclusion, high levels of MMP-9 and circulating-free DNA *BRAF^V600E^* mutation are associated with poor PFS and OS. MMP-9 may represent a promising indicator of response to BRAF inhibitors in combination with the detection of *BRAF^V600E^* mutation.

## Introduction

In the last decade, the treatment of melanoma has been revolutionized by the introduction in the therapy of several pharmacological molecules. In particular, with the recent introduction of the immune checkpoint inhibitors and the new selective tyrosine kinase inhibitors, including BRAF and MEK inhibitors, there has been a significant improvement in the progression-free survival (PFS) and overall survival (OS) of patients with melanoma (Spagnolo et al., 2016; Ugurel et al., 2017). Despite these promising results, a part of patients develops pharmacological resistance significantly reducing the effectiveness to these treatments (Sandri et al., 2016; Kalal et al., 2017). Several studies showed a very high mortality rate for cutaneous melanoma ranging from 2.17 to 2.7 per 100,000 persons (McCourt et al., 2014; Tejera-Vaquerizo et al., 2016; Brunssen et al., 2017). This high mortality rate is mainly linked to the aggressiveness of cutaneous melanomas (Mathieu et al., 2012), to the development of drug resistance (Lim et al., 2017) and to their high invasive and metastatic powers (Aladowicz et al., 2013).

It was demonstrated that CM invasiveness is mainly due to the over-expression of several proteases, including matrix metalloproteinases (MMPs) (Hofmann et al., 2000; Saladi et al., 2010; Sandri et al., 2016). In particular, a fundamental role is played by Matrix Metalloproteinase 9 (MMP-9) that promoted melanoma invasiveness and spreading via the degradation of several components of the extracellular matrix (Lu et al., 2011; Tang et al., 2013; Shi et al., 2014; Guarneri et al., 2017). Of note, the MMP-9 expression is regulated by several molecular pathways (Gordon et al., 2009; Wu et al., 2015) and its over-expression is associated to epigenetic events (Falzone et al., 2016b) and to the aberrant activation of the Ras-Raf-MEK-ERK, MAPK and PI3K/PTEN/AKT/mTOR signaling pathways (Dange et al., 2015; Shi et al., 2015). In addition, the MMP-9 expression is also regulated by several miRNAs (Falzone et al., 2016a; Yang et al., 2017).

Several point mutations affect key genes involved in the regulation of these pathways altering the cellular homeostasis resulting in cell proliferation, apoptosis prevention and tumor invasion (Zhang et al., 2016; Liu et al., 2017). Among these point mutations, *BRAF^V600E^* represents the most frequent alteration observed in melanoma (Forbes et al., 2017) and many scientific evidences suggest that *BRAF^V600E^* mutation is associated with the over-expression of MMP-9 in several tumor types, including melanoma (Mesa et al., 2006; Frasca et al., 2008; Guarneri et al., 2017). Other mutations may occur in NRAS, TERT, PTEN and, less frequently, PIK3CA (Zhang et al., 2016).

The identification of these mutated genes allowed to develop new therapeutic approaches using selective inhibitors for such altered proteins. Promising results were achieved by the treatment with BRAF inhibitors alone or in combination with MEK inhibitors (Chen et al., 2017; Russo et al., 2017). However, the identification of effective biomarker of therapeutic response is still lacking (Masucci et al., 2017; Branca et al., 2018; Ross et al., 2018; Veenstra et al., 2018).

It has been demonstrated that circulating-free DNA analysis allows to characterize the molecular features of tumors. The analysis of circulating-free DNA may be used to identify directly in serum or plasma *BRAF^V600E^* mutated clones and establish the efficacy of the treatments and the tumor aggressiveness (Schreuer et al., 2016; Herbreteau et al., 2017; Quéreux et al., 2017). However, the reduction of circulating-free DNA *BRAF^V600E^* mutated clones, followed by the treatment with BRAF inhibitors, was not directly associated with clinical efficacy (Ascierto et al., 2013; Schreuer et al., 2016). Therefore, there is a need to identify new markers that can be associated with the MAPK pathway modulation as consequence of BRAF inhibitors treatment. Among these, MMP-9 may be a right marker candidate easily detected in the peripheral blood samples from melanoma patients. Moreover, MMP-9 was demonstrated to be a marker of aggressiveness in several tumors, including melanoma (Falzone et al., 2016b; Zhang et al., 2016); while, its role as an indicator of therapeutic response was not fully investigated yet.

On these bases, in the present study functional experiments were performed using melanoma cell models to confirm the correlation between MMP-9 expression and MAPK pathway modulation during the treatment with BRAF inhibitors. Validation of *in vitro* data were assessed in peripheral blood samples from melanoma patients analyzing MMP-9 levels according to the presence of circulating-free DNA *BRAF^V600E^* mutation.

## Materials and Methods

### Cell Lines and Treatment

The A375 and A2058 melanoma cell lines were purchased from ATCC (Rockville, MD, United States). Both cell lines were cultured in RPMI-1640 medium supplemented with L-glutamine (2 mmol/L), penicillin (100 IU), streptomycin (100 μg/ml) and 10% fetal bovine serum (FBS) (all provided from GIBCO TM) and grown in humidified incubator (5% CO_2_) at 37°C. The cell lines were seeded in 60 mm cell-culture dishes (Thermo Fisher Scientific Inc., Waltham, MA, United States) at a density of 300,000 cells/well and 400,000 cells/well for A375 and A2058, respectively.

A375 cells were treated with dabrafenib (dissolved in DMSO) (cat. n. S2807 - Selleckchem, United States) at the concentration of 2, 1, 0.5, 0.25, 0.125 nM, whereas A2058 cells were treated with 32, 16, 8, 4, 2 nM of dabrafenib. DMSO was used as control. Both cell lines were treated for 12, 24, and 48 h.

Dabrafenib resistant A375 cells were obtained by culturing the cells with growing concentration of dabrafenib (up to 70 nM) for 2 months. Parental A375 and resistant A375 cells both untreated and treated with 70 nM dabrafenib were seeded in triplicate in 60 mm cell-culture dishes for 48 h.

For each cellular condition, conditioned supernatants were collected and cleaned up from debris by centrifugation. Adherent cells were collected by scraper after washing once in cold 1X DPBS GIBCO TM (cat. n. 14190250 - Thermo Fisher Scientific Inc., Waltham, MA, United States). Cell pellets were collected by centrifugation and stored at -80°C. All experiments were conducted in triplicate.

### ELISA

ELISA test was performed to detect MMP-9 levels in A375 and A2058 culture supernatants using MMP-9 Human ELISA Kit (cat. n. BMS2016-2 - Invitrogen Corporation, Carlsbad, CA, United States) according to the manufacturer’s instructions. Whereas, Human MMP-9 Quantikine ELISA KIT (cat. n. DMP900 - R&D Systems, Minneapolis, MN, United States) was used to detect serum levels of MMP-9 in melanoma patients enrolled in this study. Optical density (OD) was measured by Tecan ELISA plate reader (Tecan, Männedorf, Switzerland). Linear regression analysis was performed to generate standard curve from average absorbance of standards. MMP-9 concentrations were calculated fitting the average of duplicate ODs of each sample with standard curve.

### Real-Time PCR Quantification of MMP-9 Transcript Levels

Total RNA was extracted from cellular pellet by using the PureLink RNA mini RNA extraction kit (cat. n. 12183025 - Ambion/Life Technologies, Carlsbad, CA, United States) according to the manufacturer’s instructions. For each sample, 1 μg of total RNA was reverse transcribed using SuperScript III Reverse Transcriptase kit (cat. n. 18080044 - Thermo Fisher Scientific Inc., Waltham, MA, United States). First-strand cDNA was used for quantitative PCR (qPCR) reactions performed using the AB 7300 Real-Time PCR system (Applied Biosystems, Foster City, CA, United States). Amplification was performed using Fast SYBR Green Master Mix (cat. n. 4385617 – Applied Biosystems, Foster City, CA, United States) according to the conditions reported below:

Initial denaturation at 95°C for 10 min, followed by 40 cycles at 95°C for 15 s and 60°C for 1 min. Relative expression of MMP-9 transcript was performed using the 2^-ΔΔCt^ method. For the detection of MMP-9 transcript the following primers were used:

Forward 5′-GAACCAATCTCACCGACAGG-3′; Reverse 5′-CCACAACTCGTCATCGTCG-3′. The phosphoglycerate kinase 1 (PGK1) housekeeping gene was used as an internal control for result normalization.

PGK1 primer sequences were: Forward 5′-TTAAAGGGAAGCGGGTCGTT-3′; Reverse 5′-CAGGCATGGGCACACCAT-3′.

### Western Blot

Cellular pellets were lysed in NP-40 cell lysis buffer (cat. n. FNN0021 - Thermo Fisher Scientific Inc., Waltham, MA, United States) supplemented with protease and phosphatase inhibitor cocktails (Sigma, St. Louis, MO, United States). Quick Start^TM^ Bradford 1X Dye Reagent assay (cat. n. 5000205 - Bio-Rad Laboratories, Inc., Hercules, CA, United States) was used to quantify protein amount. For each sample, 30 μg of proteins were electrophoretically separated using 4–15% Mini Protean TGX Precast Gels (cat. n. 4561083 - Bio-Rad Laboratories, Inc., Hercules, CA, United States). Bio-Rad Trans-Blot Turbo was used to transfer the gel proteins into a PVDF/nitrocellulose membrane (Bio-Rad Laboratories, Inc.). Primary antibody Anti-MAP Kinase ERK1/ERK2 (pThr202/pThr204) rabbit Ab (diluted 1:1000 - cat. n. 442685) and Anti-MAP Kinase ERK1/ERK2 rabbit Ab (diluted 1:1000 - cat. n. 442704) (Merck Millipore, Darmstadt, Germany) were used to detect phosphorylated and total ERK 1/2 proteins.

Tubulin housekeeping protein was detected by using Anti-beta Tubulin rabbit Ab (diluted 1:1000 – cat. n. 15568- Abcam, Cambridge, United Kingdom). Chemiluminescent detection was performed using Clarity Western ECL Substrate (cat. n. 1705060 - Bio-Rad Laboratories, Inc., Hercules, CA, United States). Western blot images were collected by Bio-Rad ChemiDoc Touch Imaging System and analyzed with ImageJ software (National Institutes of Health, Bethesda, MD, United States). All Western blot experiments were performed in triplicate.

### Melanoma Patients

In this study 28 melanoma patients treated with BRAF inhibitors (53.5%) alone or in combination with MEK inhibitors (46.5%) were included. Informed consent forms were signed by each participant, and appropriate ethical committee approval was obtained. For each patient socio-demographics, clinical-pathological and therapeutics data were reported in **Table 1**. Peripheral blood samples were collected from patients prior each cycle of chemotherapy. Serum and plasma were obtained by centrifugation at 2,000 *g* for 10 min and immediately frozen (-80°C) until further analysis. All patients were enrolled at National Cancer Institute “Fondazione Giovanni Pascale,” Naples (Italy) and accepted the informed consent, according to the recommendations of the Board of Ethics of the study hospitals.

**Table 1:** Distribution of 28 patients with melanoma according to socio-demographic and clinical characteristics.

	All		Cancer progression				Vital status			
			No		Yes		Alive		Dead	
	*n*	(%)	*n*	(%)	*n*	(%)	*n*	(%)	*n*	(%)
**Sex**										
Man	14	(50.0)	2	(40.0)	12	(52.2)	4	(44.4)	10	(52.6)
Woman	14	(50.0)	3	(60.0)	11	(47.8)	5	(55.6)	9	(47.4)
Fisher test			*p* = 1.000				*p* = 1.000			
**Age at treatment initiation (years)**										
<45	11	(39.3)	2	(40.0)	9	(39.1)	5	(55.6)	6	(31.6)
45–59	10	(35.7)	1	(20.0)	9	(39.1)	1	(11.1)	9	(47.4)
≥60	7	(25.0)	2	(40.0)	5	(21.7)	3	(33.3)	4	(21.1)
Fisher test			*p* = 0.824				*p* = 0.195			
**Melanoma type**										
S.P.I.	6	(21.4)	1	(20.0)	5	(21.7)	2	(22.2)	4	(21.1)
Cutaneous	22	(78.6)	4	(80.0)	18	(78.3)	7	(77.8)	15	(79.0)
Fisher test			*p* = 1.000				*p* = 1.000			
**Stage**										
M1a	7	(25.9)	2	(20.0)	5	(22.7)	2	(22.2)	5	(27.8)
M1b	4	(14.8)	1	(10.0)	3	(13.6)	2	(22.2)	2	(11.1)
M1c	16	(59.3)	2	(20.0)	14	(63.6)	5	(55.6)	11	(61.1)
Fisher test			*p* = 0.502				*p* = 0.726			
**Therapy**									
Monotherapy	15	(57.7)	1	(20.0)	14	(60.9)	4	(44.4)	11	(57.9)
Combo	13	(42.3)	4	(80.0)	9	(39.1)	5	(55.6)	8	(42.1)
Fisher test			*p* = 0.153				*p* = 0.689
**LDH**									
<480	17	(80.0)	4	(80.0)	13	(56.5)	6	(66.7)	11	(57.9)
≥480	11	(20.0)	1	(20.0)	10	(43.5)	3	(33.3)	8	(42.1)
Fisher test			*p* = 0.620				*p* = 1.000			

### Circulating-Free DNA Isolation

Circulating-free DNA isolation was obtained from serum of melanoma samples enrolled in the present study according to manual protocols adapted from Hufnagl C and colleagues (Hufnagl et al., 2013). Briefly, 1 mL of serum, 100 μL of solution composed by EDTA (250 mmol/L) and NaCl (750 mmol/L), 100 μL of 100 g/L sodium dodecyl sulfate and 40 μL of proteinase K (stock solution 20 mg/mL) were added. After 2 h incubation at 56°C, the proteins were precipitated with 200 μL of saturated 6M NaCl solution (final concentration of 0.86 mol/L). The circulating-free DNA was extracted from supernatant by phenol-chloroform mixture at room-temperature (RT). After 5 min of incubation at RT, mixture was centrifuged for 15 min at 14,000 *g*. The upper phase was transferred in a new tube and mixed to an equal volume of absolute ethanol and then incubated over night at -20°C. The DNA was centrifuged for 15 min at 14,000 *g*. The precipitated pellet was first washed with 70% ethanol and finally dissolved in 20 μL of DNAse and RNAse free water. For two of 28 samples the extracted circulating-free DNA was found to be excessively degraded and therefore the circulating-free DNA *BRAF^V600E^* mutation detection was not performed.

### Circulating-Free DNA BRAF^V600E^ Mutation Detection

The *BRAF^V600E^* mutation was detected in circulating-free DNA using Bio-Rad droplet PCR analysis system (Bio-Rad Laboratories, Inc., Hercules, CA, United States), according to the manufacturer’s protocol. Briefly, a 22 μl of reaction mixture was prepared by mixing 11 μL ddPCR Supermix for probes (no dUTP – cat. n. 1863010), 1 μl of Bio-Rad BRAF-V600E FAM/HEX mixture (cat. n. dHsaMDV2010027), 5 μl of circulating-free DNA sample and 5 μl of PCR water. Twenty microliters of PCR reaction was loaded on the cartridge contending 70 μl of Droplet Generation Oil (cat. n. 1863005 - Bio-Rad Laboratories, Inc., Hercules, CA, United States) in appropriate wells, and then Droplet Generator QX100 was used to generate the nano-sized droplets. Droplet mixture was transferred in PCR plate and amplified by C 1000 Touch Thermal Cycler (Bio-Rad Laboratories, Inc., Hercules, CA, United States) under the following cycling conditions: 10 min at 95°C, 40 cycles of 94°C for 30 s, 55°C for 1 min, followed by 98°C for 10 min (ramp rate 2°C/s).

Fluorescent signals were detected using QX200 Droplet Reader (Bio-Rad Laboratories, Inc., Hercules, CA, United States) and analyzed using QuantaSoft software, version 1.7.4 (QuantaSoft, Prague, Czechia). Samples were considered positive when three or more FAM/HEX-positive droplets were detected. Absolute quantification (copies/mL) were determined using the QuantaSoft software.

### Statistical Analysis

MMP-9 mRNA expression, MMP-9 supernatant level and pERK/ERK ratio in treated melanoma cells and relative control were compared using two-tailed Student’s *t*-test. Fisher exact test was used to determine the association between clinical characteristics and cancer progression and vital status. Mann-Whitney test was used to compare the serum concentration of MMP-9 clustered melanoma patients according to clinical-pathological parameters.

Progression-free survival was calculated from the date of treatment initiation to progression, death, or end of follow-up, whichever occurred first. OS was calculated from the date of treatment initiation to patients’ death. Survival curves were estimated through Kaplan–Meier method; log-rank non-parametric test was used to compare the survival distributions of melanoma patients according to the detectability of circulating-free DNA *BRAF^V600E^* mutation and the MMP-9 serum concentration. Statistically significant difference was considered when *p* ≤ 0.05 (two-tailed).

## Results

### MMP-9 Expression Levels in Melanoma Cells Sensitive and Resistant to Dabrafenib

To test the hypothesis that MMP-9 expression is associated with MAPK pathway modulation, induced by the treatment with BRAF inhibitors, A375 and A2058 melanoma cells harboring *BRAF^V600E^* mutation were used.

**FIGURE 1:**
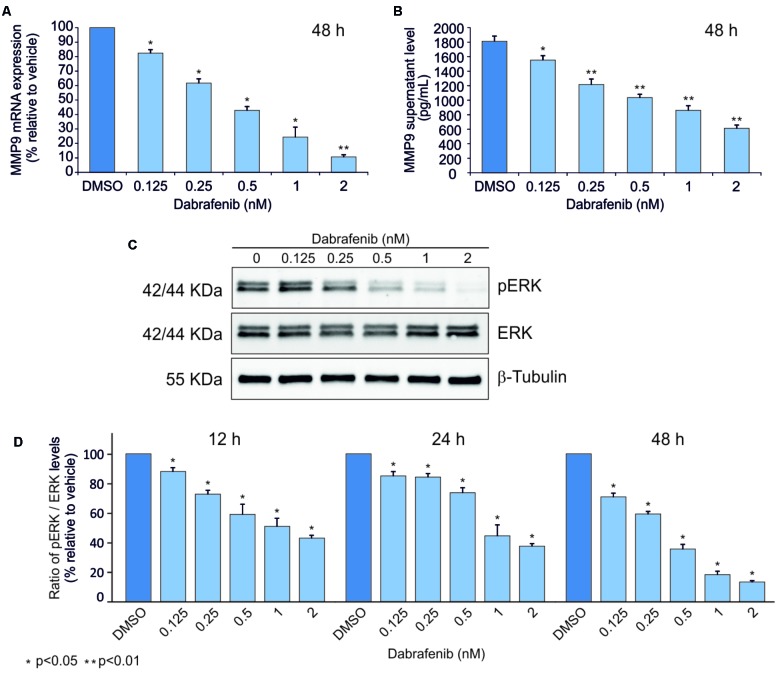
Dabrafenib treatment of A375 cell line. MMP-9 expressionlevels were evaluated by qPCR **(A)** and ELISA Assay **(B)** in A375 cell line treated with increasing doses of dabrafenib (0.125, 0.25, 0.5, 1, and 2 nM) for 48 h. pERK and total ERK protein levels were evaluated by Western blot analysis after treatment with dabrafenib for 12, 24, and 48 h **(C,D)**. The statistical significance of the two-tailed Student’s *t*-test was referred to the control. ^∗^*p* < 0.05; ^∗∗^*p* < 0.01.

**FIGURE 2:**
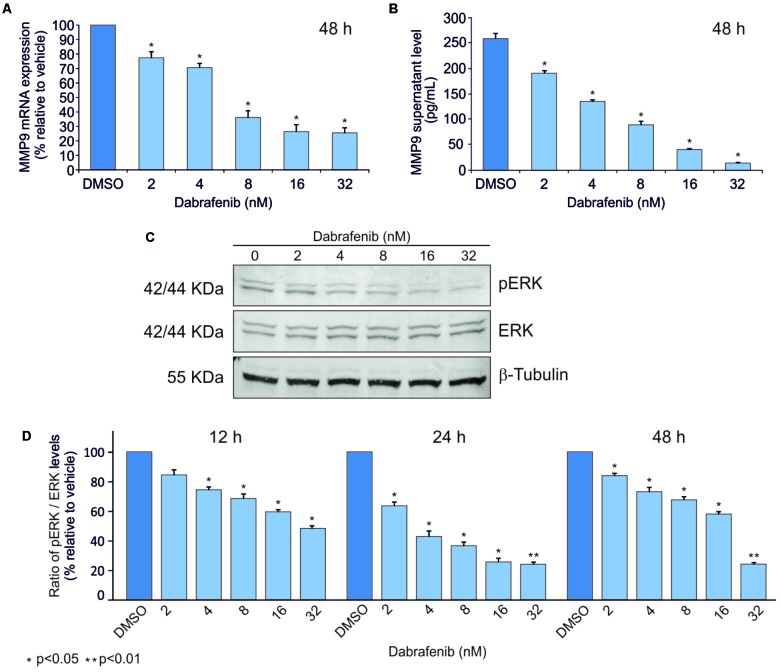
Dabrafenib treatment of A2058 cell line. A2058 cell line was treated with different doses of dabrafenib (2, 4, 8, 16, and 32 nM) for 12, 24, and 48 h. Real-Time PCR **(A)** and ELISA assay **(B)** were performed to evaluate MMP-9 expression levels of A2058 after 48 h of treatment. pERK and total ERK protein levels in A2058 cells treated with dabrafenib at 12, 24, 48 h were evaluated by Western blot **(C,D)**. The statistical significance of the two-tailed Student’s *t*-test was referred to the control. ^∗^*p* < 0.05; ^∗∗^*p* < 0.01.

ELISA and Real-Time PCR analyses showed that MMP-9 was statistically down-regulated (*p* < 0.05) in A375 and A2058 melanoma cell lines after treatment with dabrafenib for 48 h (**Figures 1A,B, 2A,B**). No significant MMP-9 modulation was observed when the cell lines were treated for 12 and 24 h (data not shown). Similar trend was observed for pERK1-2 protein levels detected by Western Blot in both A375 and A2058 cell lines at 12, 24, and 48 h (*p* < 0.05) (**Figures 1C,D, 2C,D**).

**FIGURE 3:**
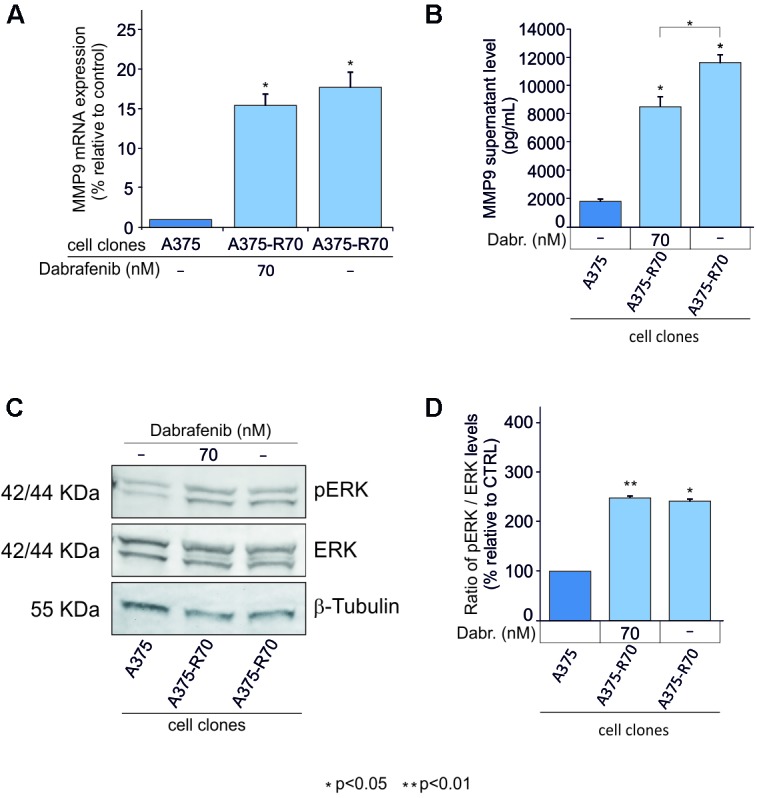
MMP-9 expression in A375 cell resistant to dabrafenib. MMP-9 expression levels were evaluated in, A375 parental clone, A375 dabrafenib resistant clones with and without addition of 70 nM of dabrafenib to RMPI-1640 medium **(A,B)**. Western Blot analysis was performed to evaluate pERK levels in resistant clones e control cell line after 48 h **(C,D)**. The statistical significance of the two-tailed Student’s *t*-test was referred to the control. ^∗^*p* < 0.05; ^∗∗^*p* < 0.01.

To verify if MMP-9 may be involved in the mechanism of resistance to BRAF inhibitors, such as dabrafenib, resistant clones of A375 were used. Both higher mRNA and protein MMP-9 expression levels were observed in the A375 resistant clones compared to those detected in the parental cells. As expected, the increase of MMP-9 expression was independent to the treatment with dabrafenib (**Figures 3A,B**). To further investigate the effects of resistance to dabrafenib on MAPK pathway, pERK1-2 levels in resistant clones were measured. pERK1-2 levels were higher in resistant clones when compared with sensitive parental cells (*p* < 0.05) (**Figures 3C,D**).

These *in vitro* data showed that BRAF inhibitor efficiently counteracts the MAPK signal pathway in A375 cells where the concomitant decrease of pERK and MMP-9 levels was observed in a dose-dependent manner. In contrast, activation of pERK was observed in resistant cell clones with concomitant MMP-9 overexpression. These encouraging data support the notion that MMP-9 may be considered a marker of response to dabrafenib in melanoma cells.

### Circulating MMP-9 Expression Levels in Peripheral Blood From Melanoma Patients Treated With BRAF Inhibitors

**FIGURE 4:**
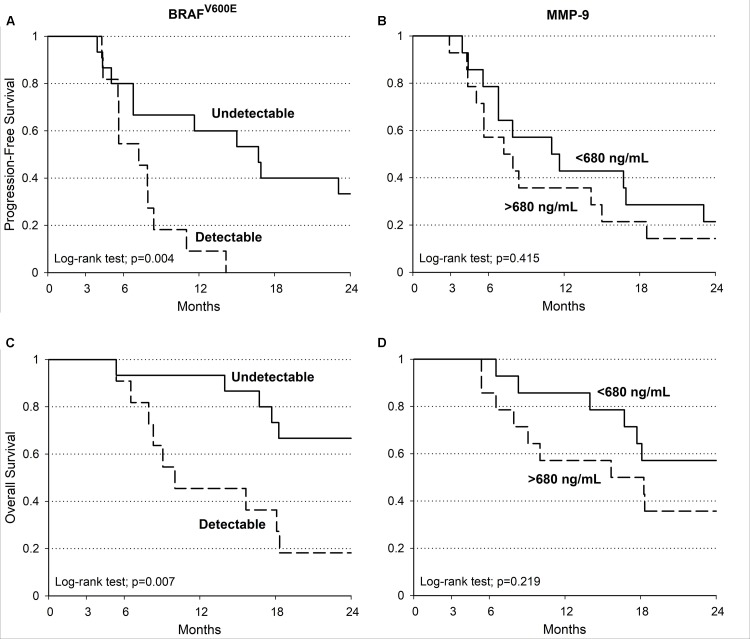
Melanoma patients PFS and OS. Kaplan–Meier estimate of PFS and OS in patients with detectable (dotted line) and undetectable (continuous line) *BRAF^V600E^* (PFS log-tank test: *p* = 0.004; OS log-tank test: *p* = 0.007) **(A,C)**. PFS and OS evaluations in patients with MMP-9 serum concentration at baseline (continuous line: < 680 ng/mL; dotted line: ≥ 680 ng/mL; PFS log-tank test: *p* = 0.415; OS log-tank test: *p* = 0.219) **(B,D)**.

MMP-9 serum levels were evaluate in melanoma samples according to socio-demographic, clinical and molecular features, including the presence of circulating-free DNA *BRAF^V600E^* mutation (**Table 2**). Such mutation was identified in 11 out of 26 melanoma patients (42%). Notably, MMP-9 higher levels were observed in patients with detectable circulating-free DNA *BRAF^V600E^* mutation compared to those with undetectable *BRAF^V600E^* mutation (*p* = 0.058). In agreement with these data, it appears speculating that higher levels of MMP-9 are associated with the spreading of tumor DNA in the bloodstream where *BRAF^V600E^* mutation is detectable (**Table 2**). Accordingly, the PFS and OS are higher in melanoma patients with undetectable circulating-free DNA *BRAF^V600E^* mutation compared with those harboring the detectable mutation (*p* = 0.004 and *p* = 0.007 respectively) (**Table 3** and **Figures 4A,C**). These data indicate that the detectability of circulating-free DNA *BRAF^V600E^* mutation may be considered as a negative prognostic factor. Although no statistics significance has been reached due to the low number of patients, it was observed that highest MMP-9 levels were associated with a lower PFS and OS (**Table 3** and **Figures 4B,D**). No statistical difference was observed for both PFS and OS in patients treated with monotherapy or combination therapy (**Table 3**).

**Table 2:** MMP-9 concentration at baseline in serum of 28 patients with melanoma according to socio-demographic, clinical characteristics and molecular features.

	*n*	MMP-9 (ng/mL)		Mann–Whitney test
		Median	Interquartile range	
Overall		684	427–1118	
**Sex**				
Man	14	684	417–1108	*p* = 0.927
Woman	14	662	437–1128	
**Age at treatment initiation (years)**				
<45	11	601	482–1148	*p* = 0.807
45–59	10	870	352–882	
≥60	7	670	354–1108	
**Melanoma type**				
S.P.I.	6	1204	417–1930	*p* = 0.131
Cutaneous	22	636	437–873	
**Stage**				
M1a	7	601	482–723	*p* = 0.624
M1b	4	614	470–1801	
M1c	16	872	427–1159	
**Therapy**				
Monotherapy	15	697	437–1108	*p* = 0.596
Combo	13	670	352–1128	
**LDH**				
<480	17	671	482–882	*p* = 0.796
≥480	11	686	354–1238	
****BRAF*^*V600E*^***				
Undetectable	15	557	354–722	*p* = 0.058
Detectable	11	873	437–1238	

**Table 3:** Median time to progression (TTP) and progression free survival (PFS) in 28 patients with melanoma according to BRAF and MMP-9.

	Patients	Progressions	Median TTP (days)	PFS			Deaths	OS		
				6 mos	12 mos	24 mos		6 mos	12 mos	24 mos
Overall	28	23	219	67.9%	39.3%	17.9%	14	92.9%	71.4%	46.4%
***BRAF**^**V600E**^*										
Undetectable	15	10	280	80.0%	60.0%	33.3%	5	93.3%	93.3%	66.7%
Detectable	11	11	219	54.6%	9.1%	0.0%	9	90.9%	45.5%	18.2%
			*p* = 0.398	Log-rank test: *p* = 0.004				Log-rank test: *p* = 0.007		
**MMP-9 at treatment initiation (ng/mL)**										
<680	14	11	240	78.6%	42.9%	21.4%	6	100%	85.7%	57.1%
≥680	14	12	195	57.1%	35.7%	14.3%	9	85.7%	57.1%	35.7%
			*p* = 0.450	Log-rank test: *p* = 0.415				Log-rank test: *p* = 0.219		
***BRAF**^**V600E**^* **and MMP-9 at treatment initiation (ng/mL)**										
Undetectable and <680	10	7	353	90.0%	60.0%	30.0%	3	100%	100%	70.0%
Undetectable and ≥680	5	3	153	60.0%	60.0%	40.0%	2	80.0%	80.0%	60.0%
Detectable and <680	4	4	205	50.0%	0.0%	0.0%	3	100%	50.0%	25.0%
Detectable and ≥680	7	7	219	57.1%	14.3%	0.0%	6	85.7%	42.9%	14.3%
			*p* = 0.153	Log-rank test: *p* = 0.037				Log-rank test: *p* = 0.056		
**Therapy**										
Monotherapy	15	14	206	60.0%	26.7%	0.0%	9	100%	60.0%	40.0%
Combo	13	9	240	76.9%	53.9%	30.8%	6	84.6%	84.6%	53.9%
			*p* = 0.705	Log-rank test: *p* = 0.102				Log-rank test: *p* = 0.496		

**FIGURE 5:**
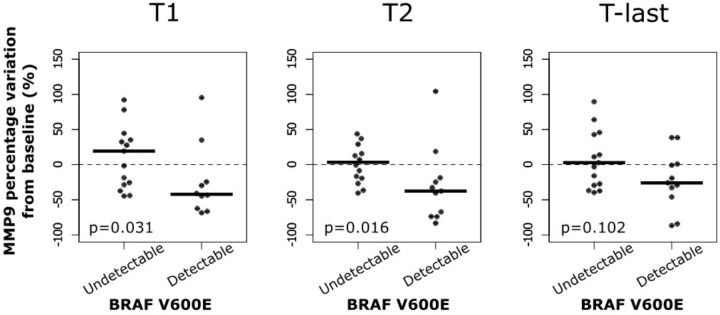
MMP-9 reduction in patients with *BRAF^V600E^* mutation at different time. Percentage variation of MMP-9 from baseline to first (T1) and second (T2) clinical examination after treatment initiation and to last follow-up (T-last) according to circulating *BRAF^V600E^* mutation.

In addition, the percentage of MMP-9 serum variations at different times during the treatment with BRAF inhibitors were analyzed in melanoma patients. Such variations were also evaluated according to the detectability of circulating-free DNA *BRAF^V600E^* mutation. No significant percentage variations of MMP-9 serum levels were observed in melanoma patients at different times during the treatment with BRAF inhibitors (data not shown). While, a significant percentage of MMP-9 decrement in samples with circulating-free DNA *BRAF^V600E^* mutation compared to those with undetectable one was observed at T1 and T2 (**Figure 5**). Such percentage of MMP-9 decrement was not observed at T-last where novel microenvironmental factors and/or genetic alteration may occur conferring a drug resistance phenotype to the tumor cells (**Figure 5**). These data indicate that the MMP-9 decrement is more evident during the first week of treatment, where the therapy is effective, in melanoma patients with detectable circulating-free DNA *BRAF^V600E^* mutation. However, at last follow-up MMP-9 decrement in those patients is not significant compared to patients with undetectable *BRAF^V600E^* mutation, suggesting that the treatment became ineffective.

## Discussion

In the last decade, the treatment with BRAF and MEK kinase inhibitors improved the prognosis of advanced melanoma. However, a significant fraction of these patients experiences progression disease. The identification of new biomolecular markers of progression disease and acquired resistance may lead to change therapeutic setting and, eventually, reduce overtreatment (Fattore et al., 2016, 2017; Leonardi et al., 2018).

Improvement of liquid biopsy through the recent advances in molecular technologies, such as the development of digital polymerase chain reaction (dPCR), led to identify tumor molecular features by analyzing the circulating-free DNA harboring pathogenetic mutations (Busser et al., 2017; Gorgannezhad et al., 2018). Several studies showed that detection of circulating-free DNA *BRAF^V600E^* mutations is predictive of the response to MAPKs inhibitors in melanoma treatment. In particular, high basal levels of circulating-free DNA *BRAF^V600E^* mutation are associated with poor response to therapy and PFS and OS (Ascierto et al., 2013; Sanmamed et al., 2015; Santiago-Walker et al., 2016; Lee et al., 2018). Furthermore, it has been demonstrated that increase of circulating-free DNA BRAF mutation during treatment is predictive of acquired resistance and therapeutic failure (Gray et al., 2015).

To the best of our knowledge, no previous studies have identified new markers that can be linked with the MAPK pathway modulation after treatment with BRAF inhibitors. However, MMP-9 has been associated with the aberrant activation of MAPK pathway in melanoma, suggesting its role as prognostic indicator (Guarneri et al., 2017). Therefore, in the present study, the modulation of MMP-9 in melanoma after treatment with B-RAF inhibitors was analyzed through different experimental approaches.

Our *in vitro* data showed that BRAF inhibitor efficiently counteracts the MAPK signal pathway in A375 cells where the concomitant decrease of pERK and MMP-9 levels was observed in a dose-dependent manner. Furthermore, to verify if MMP-9 may be involved in the mechanism of resistance to BRAF inhibitors A375 resistant cells were obtained and pERK protein levels showed opposite behavior compared to sensitive cells when treated with dabrafenib. In particular, an activation of pERK was observed in resistant cell clones with concomitant MMP-9 overexpression. These encouraging data support the notion that MMP-9 may be considered a marker of response to dabrafenib in melanoma cells.

After the *in vitro* evaluations, circulating-free DNA *BRAF^V600E^* mutation and MMP-9 serum levels were evaluated in melanoma patients. Unexpectedly, circulating-free DNA *BRAF^V600E^* mutation was detected in only the 42% (11 out of 26) of melanoma patients positive to *BRAF^V600E^* mutation after the histopathological diagnosis. Interestingly, ELISA assay showed that MMP-9 serum levels where higher in patients with detectable circulating-free DNA *BRAF^V600E^* mutation compared to those with undetectable *BRAF^V600E^* mutation suggesting that the increase of MMP-9 in the subset of patients with detectable circulating mutation are associated with the spreading of tumor and in the release of circulating-free DNA in the bloodstream. These evidences encourage the use of MMP-9 as a prognostic marker in the subsets of patients with detectable circulating mutation, where the concomitant evaluation of circulating-free DNA status and MMP-9 serum levels may give important information about the prognosis of patients.

Indeed, the PFS and OS analyses according to the circulating-free DNA *BRAF^V600E^* status and the MMP-9 expression levels showed a worse prognosis for melanoma patients with detectable circulating *BRAF^V600E^* mutation and high MMP-9 serum levels (>680 ng/mL) compared with those with undetectable mutation and lower levels of MMP-9.

Finally, the analysis of MMP-9 during the treatment showed that MMP-9 serum protein levels significantly decreased at T1 and T2, but not at the last follow-up, for the patients with detectable circulating-free DNA *BRAF^V600E^* mutation.

Overall, the results of the present study confirm that the occurrence of circulating-free DNA *BRAF^V600E^* mutation is a negative prognostic factor of cutaneous melanoma; furthermore, MMP-9 may be considered a prognostic indicator of response to BRAF inhibitors only in the subset of patients harboring the circulating-free DNA *BRAF^V600E^* mutation supporting the notion that MMP-9 is associated with the MAPK pathway. Further studies, including a larger series of melanoma patients, are needed to better clarify the impact of MMP-9 as a marker of response to treatment with BRAF inhibitors.

## Ethics Statement

This study was carried out in accordance with the recommendations of Ethics Committee of the Istituto Nazionale Tumori of Naples with written informed consent from all subjects. All subjects gave written informed consent in accordance with the Declaration of Helsinki. The protocol was approved by the Ethics Committee of the Istituto Nazionale Tumori of Naples.

## Author Contributions

SC, ML, and PA conceived the study. SC and GM dealt with the study design in all its experimental phases. LF, RS, and DC performed the experiments and the quality control of data and procedures. GM, DM, LF, and RS performed the data analyses and interpretation, while, JP executed all the statistical analyses. LF and SC wrote the manuscript. ML and PA reviewed the final version of the manuscript. All authors read and approved the final version of the manuscript.

## Conflict of Interest Statement

The authors declare that the research was conducted in the absence of any commercial or financial relationships that could be construed as a potential conflict of interest.
